# A Case of Tuberculosis-related Leukocytoclastic Vasculitis Presenting With Peripheral Neuropathy

**DOI:** 10.7759/cureus.3703

**Published:** 2018-12-07

**Authors:** Nastaran Rafiei, Negar Khanlou, Shaweta Khosa, Negar Moheb, Shri K Mishra

**Affiliations:** 1 Neurology, David Geffen School of Medicine at University of California, Los Angeles, USA; 2 Pathology, David Geffen School of Medicine at University of California, Los Angeles, USA; 3 Neurology, Olive View - University of California Los Angeles Medical Center, Los Angeles, USA; 4 Neurology, Keck School of Medicine of the University of Southern California, Los Angeles, USA

**Keywords:** tuberculosis, allergic vasculitis, leukocytoclastic vasculitis, neuropathy

## Abstract

Tuberculous granulomatous vasculitis is commonly associated with meningitis and retinitis. We describe a 39-year-old male, with a history of pulmonary tuberculosis (TB) who presented with progressive weakness, pain, tingling and numbness in the bilateral lower extremities. Significant atrophy and weakness of the lower extremities were evident along with absent reflexes. Nerve conduction studies and electromyography showed severe axonal polyneuropathy and denervation on the lower extremities. Nerve biopsy demonstrated small vessel leukocytoclastic vasculitis without any granuloma formation. Muscle biopsy was consistent with denervation and atrophy with target fiber changes. Tuberculosis-related vasculitis causing peripheral neuropathy is extremely rare and our case is unique in manifesting this presentation.

## Introduction

Tuberculosis (TB) has been one of the world's most prevalent communicable diseases. The number of TB cases is slowly declining every year. According to a report published by the World Health Organization (WHO), an estimated 10.4 million people developed TB and 1.6 million died from the disease in 2016 [1]. Tuberculosis has been reported to have a variety of clinical manifestations, and TB-related vasculitis has been one of the rare ones.

Vasculitis can affect blood vessels of all sizes in any organ, and this results in a wide variety of signs and symptoms in the clinical presentation [2]. Vasculitis can be classified in many ways: 1) localized: such as isolated cutaneous vasculitis, or 2) systemic: affecting various organs and systems. It can also be classified based on the etiology, as primary (idiopathic) or secondary within the context of another pathological process, such as autoimmune diseases like systemic lupus erythematosus, neoplasms, drug hypersensitivity, systemic vasculitic disorders such as Wegener’s disease and both viral and bacterial infections. Infection can cause vascular damage not only by direct invasion of the vessel wall but also by immune complex deposition or through secondary cryoglobulinemia [3]. Systemic vasculitis causing peripheral neuropathy with pulmonary involvement can occur in granulomatosis with polyangiitis, eosinophilic granulomatosis with polyangiitis (EGPA), polyarteritis nodosa, and microscopic polyangiitis. Tuberculous granulomatous vasculitis, on the other hand, is commonly associated with meningitis and retinitis [4-6].

## Case presentation

We describe a 39-year-old male with a diagnosis of pulmonary tuberculosis who presented with progressive asymmetric weakness, pain, tingling and numbness in the lower extremities over the past year. The patient was diagnosed with pulmonary tuberculosis five years ago and was treated for a total duration of 26 months for multidrug resistant (MDR) TB. His past medical history was significant for vitamin B12 deficiency and a deep vein thrombosis of the left femoral vein extending down to the popliteal vein.

**Figure 1:**
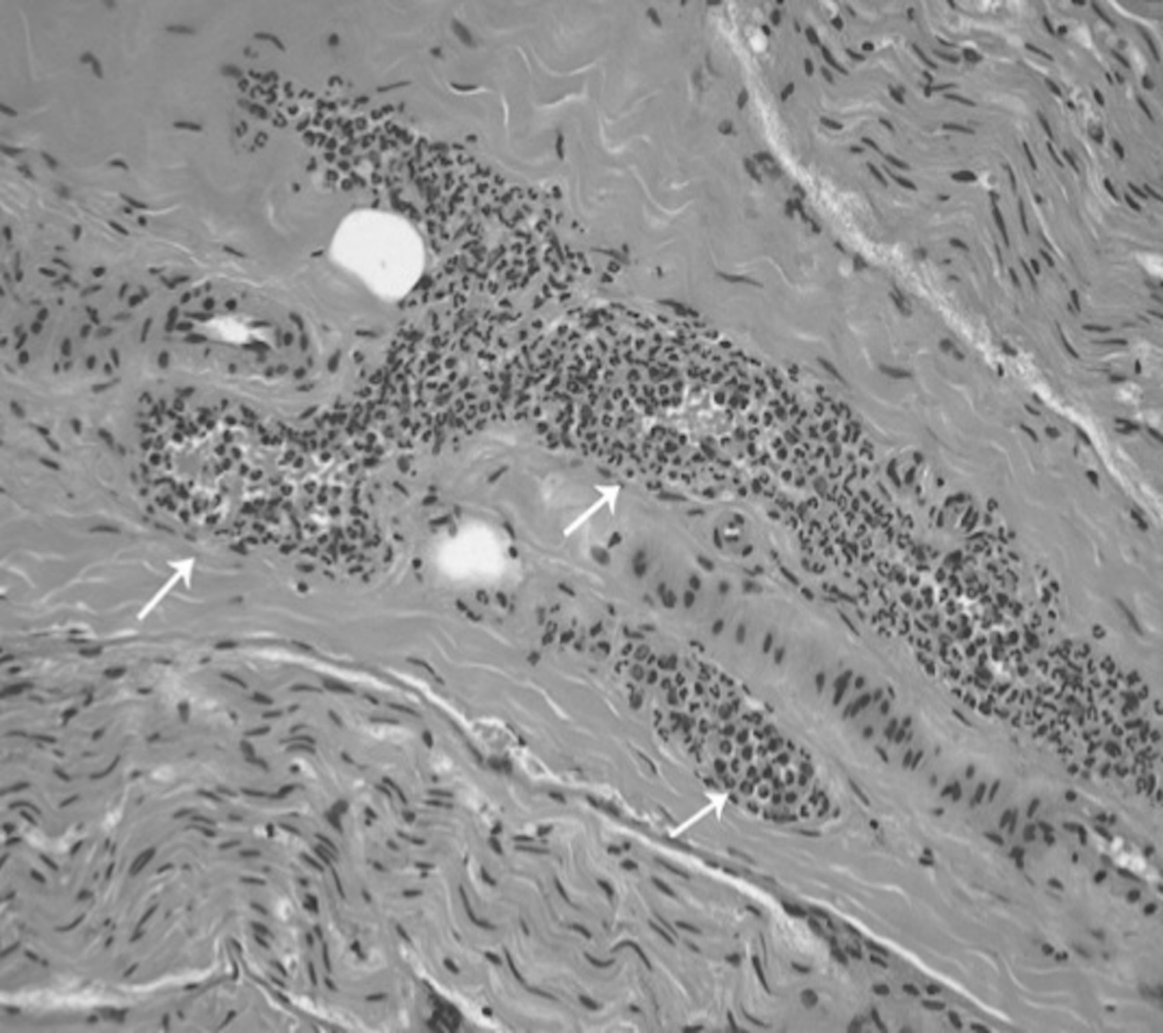
Peripheral nerve biopsy (paraffin hematoxylin & eosin X10) Leukocytoclastic vasculitis with obliteration of vessel wall by leukocytes and eosinophils (white arrows)

**Figure 2:**
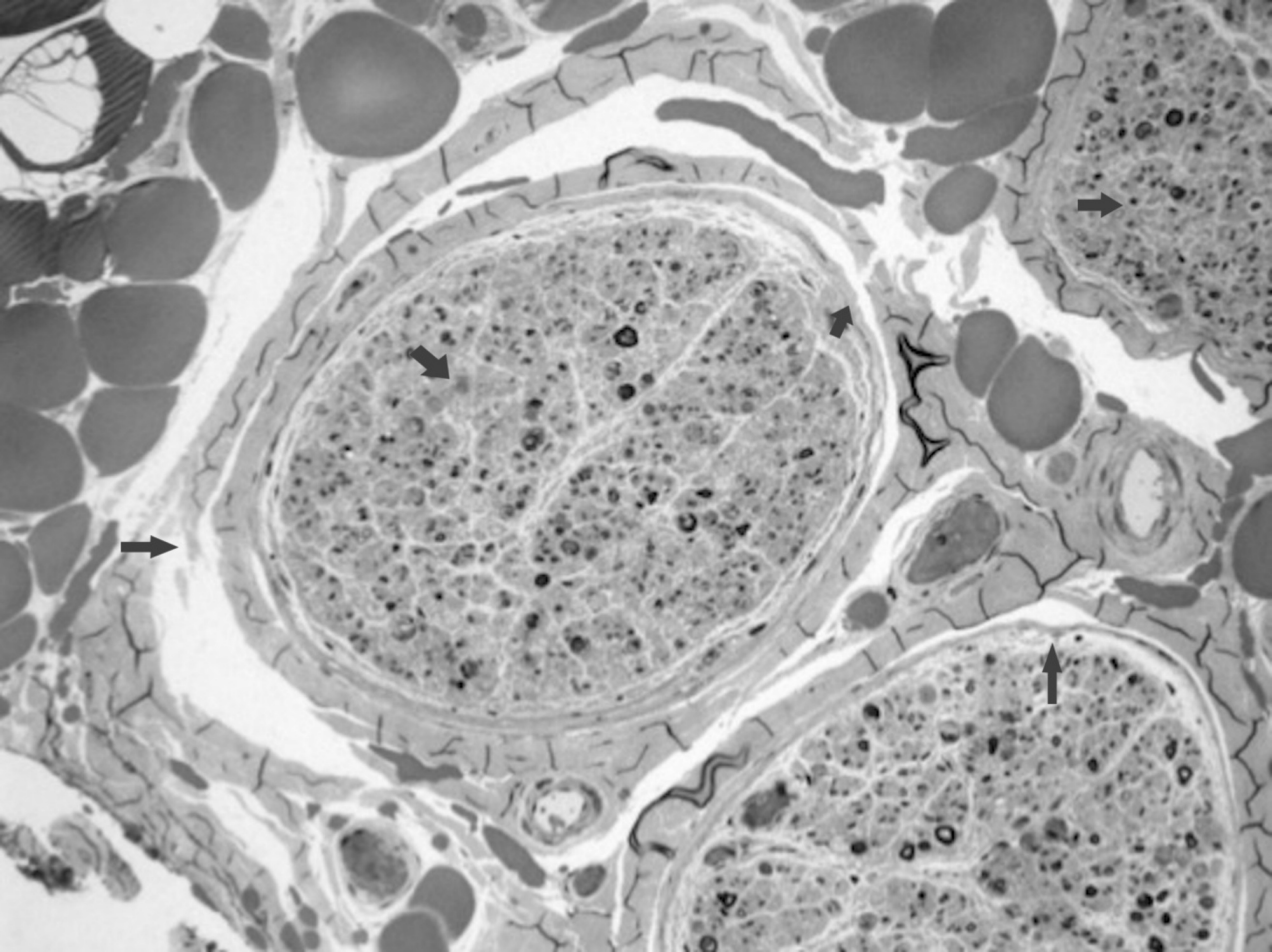
Peripheral nerve (methylene blue stained one micron resin section X 4) Portions of three fascicles with myelinated fiber loss and active degeneration/demyelination

On examination, lower extremity reflexes were absent. Significant atrophy and severe weakness of the lower extremities was evident. The sensory examination was significant for marked hyperesthesia and loss of pinprick and proprioception sensation of the lower extremities. The patient had a positive QuantiFERON-TB Gold test (QIAGEN Inc., MD, USA). His sputum was positive for acid fast bacilli (AFB) on presentation. Chronic inflammation markers including erythrocyte sedimentation rate (ESR) and C-reactive protein (CRP) were elevated. Nerve conduction studies showed evidence of severe axonal sensorimotor polyneuropathy. Electromyography (EMG) suggested acute and chronic denervation of all the lower extremity muscle groups with normal findings in the upper extremities. A biopsy of the sural nerve demonstrated small vessel leukocytoclastic (allergic) vasculitis with intrusion of the epineural and perineurial vessel walls by leukocytes and eosinophils, vascular luminal obstruction, and resulting considerable active Wallerian/axonal degeneration. There was no granuloma formation as seen in Figures 1-2. A gastrocnemius muscle biopsy showed significant angular fiber atrophy with target fiber changes, consistent with acute/sub-acute denervation.

Workup for other etiologies resulting in vasculitis such as Wegener's disease, autoimmune/connective tissue disorders, cryoglobulinemia and HIV-related vasculitis were all negative. The patient was then treated with anti-tuberculosis medications and immune modulating agents (steroid and Rituxan). There was modest improvement of the weakness in his lower extremities.

## Discussion

Small-vessel vasculitis refers to a wide range of diseases characterized by inflammation of the walls of venules, capillaries, and/or arterioles with a variety of clinical presentations [7]. The classical clinical phenotype is leukocytoclastic vasculitis (LCV) also known as hypersensitivity vasculitis, and hypersensitivity angiitis shows palpable purpura, but manifestations vary widely depending upon the organs involved. Histopathologically, LCV is characterized by an angiocentric inflammatory process associated with leukocytoclasia (neutrophil fragmentation), fibrinoid necrosis, and a neutrophilic infiltrate around the blood vessel walls with erythrocyte extravasation. The etiology of small-vessel vasculitis is unknown in many cases, but it can be secondary to drugs, infections, malignancy, primary vasculitis such as microscopic polyarteritis, and connective tissue disorders [8].

Neuropathy as a result of allergic vasculitis is extremely rare in the case of tuberculosis [9,10]. Cutaneous leukocytoclastic vasculitis has been reported previously to accompany tuberculosis including pulmonary tuberculosis, tubercular lymphadenitis, and multifocal tuberculosis [9,11]. The exact mechanism of cutaneous leukocytoclastic vasculitis is still not known. Various possible mechanisms proposed include immunogenic reaction to the bacilli, deposition of immune complexes, direct invasion by tubercular bacilli in the vessels, delayed type hypersensitivity reaction as well as with the use of anti-tuberculosis medications [12-14]. Previous case reports have reported pulmonary tuberculosis and vasculitis in association with cutaneous leukocytoclastic vasculitis, Henoch-Schönlein purpura, and vasculitis secondary to anti-tubercular treatment but none of them have reported peripheral neuropathy. Our case is unique in reporting a clinical presentation of neuropathy in a patient with tuberculosis and identification of concomitant vasculitic injury in the biopsy material. The patient presented with the symptoms of vasculitis and neuropathy, which improved while on anti-tubercular regimen making it less likely related to medication. The workup for various other possible etiologies for vasculitis and neuropathy was negative. The timeline of presentation and the vasculitis confirmed on biopsy findings in a patient with tuberculosis makes tuberculosis-related vasculitis likely the cause of peripheral neuropathy. In this report we described a unique case of histology-confirmed tuberculosis associated with LCV presenting with peripheral neuropathy. However, the exact pathogenesis and other etiologies of the peripheral neuropathy is worthy of investigation.

## Conclusions

We describe a rare case of peripheral neuropathy caused by tuberculosis-related vasculitis. It is a unique manifestation of tuberculosis, rarely reported and likely secondary to immunological reactions to tubercle bacilli and/or rifampicin with antibodies and immune complex formation. Further investigations are needed to determine the exact etiology and potential role of anti-tuberculosis medication.
